# Spatial patterns of childhood diarrhea in Ethiopia: data from Ethiopian demographic and health surveys (2000, 2005, and 2011)

**DOI:** 10.1186/s12879-017-2504-8

**Published:** 2017-06-15

**Authors:** Getahun Gebre Bogale, Kassahun Alemu Gelaye, Degefie Tibebe Degefie, Yalemzewod Assefa Gelaw

**Affiliations:** 1 Department of Basic Health Sciences, Dessie Health Science College, Dessie, Ethiopia; 2 Department of Epidemiology and Biostatistics, Institute of Public Health, College of Medicine and Health Sciences, University of Gondar, Gondar, Ethiopia; 3 Department for Landscape Ecology, Research Scientist, University Münster, Working Group Climatology, Münster, Germany

**Keywords:** Childhood, Cluster, Diarrhea, Pattern, Spatial, Ethiopia

## Abstract

**Background:**

Childhood diarrhea is a major public health problem, especially in developing countries, including Ethiopia. Exploring the spatial pattern of childhood diarrhea is important to monitor and design effective intervention programs. Therefore, this study aimed to explore the spatial patterns of childhood diarrhea in Ethiopia over the past one decade.

**Methods:**

A total of 29,358 under-five children were retrieved from three consecutive Ethiopian demographic and health surveys (2000, 2005, and 2011) and included into the study. Spatial cluster and autocorrelation analysis was done to explore the patterns of childhood diarrhea.

**Results:**

Childhood diarrhea clustered spatially at a national level in all survey periods (Moran’s I: 0.3830–1.3296, *p* < 0.05). Significant spatial clusters were found in different survey periods across the regions. The most likely spatial clusters were found in Southern Nations Nationalities and people, West Oromia, Gambella, Benshangul-Gumuz, and Somali regions. Childhood diarrhea also clustered at the border areas of Southern Nations Nationalities and People and Tigray, Central Somali and Western Oromia, Gambella and Amhara (West Gojam, Awi, Oromia, and Wag Himra) regions. In 2000, the most likely clusters were found in Southern Nations Nationalities and People, West Oromia, and Gambella regions (LLR = 55.37, *p* < 0.001); in 2005, at Southern Nations Nationalities and People (LLR: 45.69, *p* < 0.001); and in 2011, at Gambella, West Southern Nations Nationalities and People and Oromia, and Benshangul-Gumuz regions (LLR: 51.09, *p* < 0.001).

**Conclusion:**

In this study, childhood diarrhea remains public health problem and had a spatial variation across the regions. Identifying the risk areas would help in designing effective interventions to reduce childhood diarrhea in these areas.

## Background

Worldwide, childhood diarrhea remains a leading cause of morbidity and mortality. Each year, around 800,000 children died due to diarrheal disease mostly in developing countries and sub-Saharan Africa including Ethiopia [1].

Studies showed childhood diarrhea has variation and varied geographically [2–4]. A study done in Thailand revealed that diarrhea has a spatially clustered pattern [5]. Studies in Africa exhibits the prevalence of childhood diarrhea varied in geographically [6, 7].

In Ethiopia, many public health efforts have been performing across the country to prevent infectious diseases including diarrhea. Prevention and control of childhood diarrhea is under the package of family health services in the Health Extension Program, a health work forces at the peripheral health units launched in 2004 [8]. However, childhood diarrhea remains the second common cause of under-five mortality and morbidity in the country [9]. Previous researches done in different parts of Ethiopia reported that prevalence of diarrhea marks highly variability at the study setting levels. Socio-demographic factors, personal factors, and environmental factors are thought to be associated factors of diarrhea prevalence [10–14]. This continuing problem in the country varies across the time by changing the disease burden. Whereas, the country lacks identifying risk areas of diarrhea transmission [2]. ArcGIS® and SaTScan™ based statistical techniques are widely used to analyze the spatial patterns of disease and to identify the high-risk hotspots [15–17].

This study aimed to explore the spatial patterns of childhood diarrhea in Ethiopia over the past one decade. Thus, these findings would be essential to provide geographic-based evidence for decision makers and collaborators to formulate strategies and act appropriately.

## Methods

### Study design and setting

**Fig. 1:**
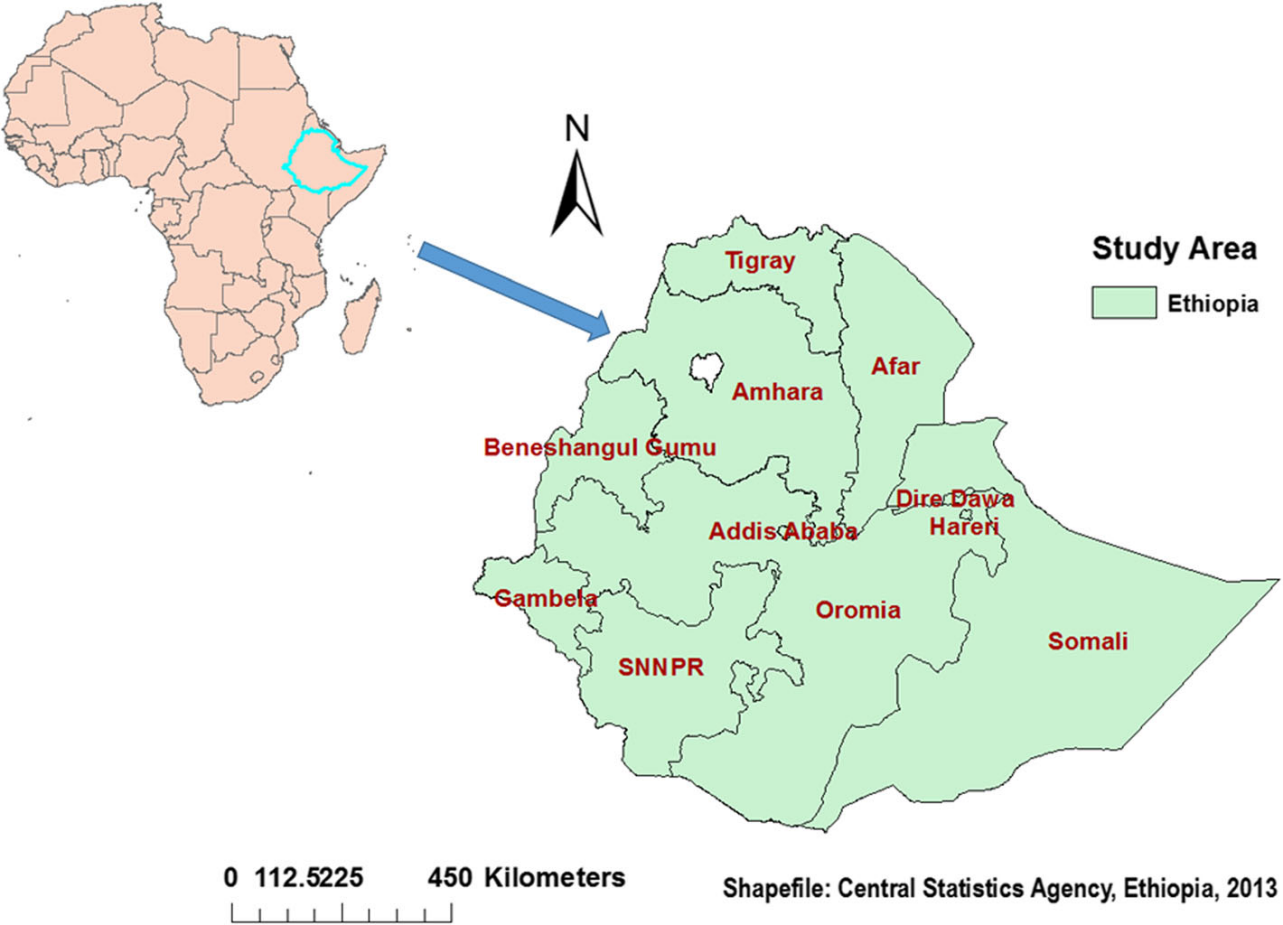
Map of Ethiopia where the study is undertaken, 2013. The map shows geographical locations of nine regional states and two city administrations as labelled by their names

Population-based repeated cross-sectional study was employed to identify the extent of spatial patterns of childhood diarrhea in Ethiopia: Data from Ethiopia DHS 2000, 2005, and 2011. The study is conducted in Ethiopia (3^o^ - 14^o^ N and 33^o^ - 48°E), situated at the eastern tip of Africa (Fig. 1). The country covers 1.1 million Sq. km and has a great geographical diversity, which ranges 4550 m above sea level down to the Afar depression to 110 m below sea level. There are nine regional states and two city administrations subdivided into 68 zones, 817 districts and 16,253 *kebeles* (*lowest local administrative units of the country*) in the administrative structure of the country [9].

### Data source and measurement

In every five years, the Demographic and Health Survey program of the country (EDHS) has collected data on national representative samples of all age group and key indicators including childhood diarrhea. There were 16.9% (11.3 million), 15.8% (10.7 million), and 15.4% (11.75 million) of under-five years of age children were recorded in 2000, 2005, and 2011 surveys, respectively (Table 1) [9, 18, 19]. The questionnaire included sociodemographic, socioeconomic, child health and maternal related variables towards childhood diarrhea.

**Table 1:** Total number of under five children included in 2000, 2005, and 2011 EDHS, Ethiopia

Years of Surveys	Sample size (n)		
	Urban	Rural	Total
2000	1536	8012	9548
2005	1275	7727	9002
2011	1865	8943	10,808
Total	4676	24,682	29,358

A stratified two-stage cluster sampling procedure was employed for all the three surveys. In 2000 and 2005 surveys, 540 enumeration areas (EAs) (139 urban and 401 rural areas) were selected using systematic sampling with probability proportional to size. A total of 14,642 households and 15,716 eligible women, and 14,645 households and 14,717 eligible women with their under-five children diarrhea status, respectively, were included. In 2011 EDHS, 624 EAs (187 urban and 437 rural areas) were selected. Of these, 17,817 households and 17,385 eligible women with their under-five children were included in the study. The occurrence of diarrhea were categorized as “Yes” or “No” by asking the caregivers/mothers whether their children had experienced diarrhea in the last two weeks preceding the survey [9, 18, 19].

Location data (latitude and longitude coordinates) were also taken from selected enumeration areas. The survey datasets and location data were accessed through the web page of International DHS Program after subscription and being an authorized user [20].

Descriptive and summary statistics for non-spatial analysis was done using STATA version 12 software. Geographical Information System (ArcGIS version 10.1) software was used to analyze spatial statistics. Childhood diarrhea defined as the frequent (three or more times per day) passage or loss of liquid stools within two weeks period prior to survey [21, 22].

### Statistical analysis

A global and local scale spatial autocorrelation analysis were used to explore the presence of clustering in the area and detect the geographical location of clusters of childhood diarrhea. The global Moran’s *I statistic* was used to measure the geographical clustering over the nation. Whereas Local Moran’s *I* used to indicate the local clustering and to identify the locations of hotspots, however, it has less power than others [15]. Multiple tests were used to show the consistency of findings, as they are used by other relevant studies [23, 24].

### Spatial autocorrelation analysis

The spatial autocorrelation (Global Moran’s *I*) statistic measures were used to evaluate whether the disease patterns are dispersed, clustered or randomly distributed in the study area [5]. It was used to detect the spatial autocorrelation of diarrhea: calculated Moran’s *I* values close to −1 indicate disease dispersed, whereas *I* close to +1 indicate disease clustered and disease distributed randomly if *I* value zero. A statistically significant Moran’s I (*p* < 0.05) leads to rejection of the null hypothesis and indicates the presence of spatial autocorrelation [25].

Anselin Local Moran’s *I* was used to investigate the local level cluster locations of diarrhea. Local Moran’s *I* measures whether there were positively correlated (high-high and low-low) clusters or negatively correlated (high-low and low-high) clusters of high values (High-High), clusters of low values (Low-Low). It also measures outlier in which a high value is surrounded primarily by low values, and outlier in which a low value is surrounded primarily by high values [26, 27]. A positive value for ‘*I*’ indicated that a case had neighboring cases with similar values, such type of case was part of a cluster. A negative value for *‘I’* indicated that a case was surrounded by cases with dissimilar values; this case was an outlier [5].

### Hot spot analysis (Getis-Ord Gi* statistic)

Gettis-Ord Gi* statistics was computed to measure how spatial autocorrelation varies over the study location by calculating Gi* statistic for each area. Z-score is computed to determine the statistical significance of clustering, and the *p*-value computed for the significance [25]. The *p*-value associated with a 95% confidence level is 0.05. If the z-score is between −1.96 and +1.96, the *p*-value would be larger than 0.05, and could not reject the null hypothesis; the pattern exhibited could very likely be the result of random spatial processes. If the z-score falls outside the range, the observed spatial pattern is probably too unusual to be the result of random chance, and the *p*-value would be small to reflect this. In this case, it is possible to reject the null hypothesis and proceed with figuring out what might be causing the statistically significant spatial pattern in the data. Statistical output with high Gi* indicates “hotspot” whereas low Gi* means a “cold spot” [26, 28, 29].

### Spatial interpolation

Spatial interpolation technique was applied to predict the unsampled from sampled measurements. Kriging spatial interpolation method used for predictions [28] and produce smooth surfaces [16, 30] of childhood diarrhea. Therefore, ordinary kriging was used to estimate the burden of diarrhea in this study [31].

### Disease cluster detection and spatial scan statistical analysis

Spatial Scan statistical method is widely recommended that it performs very well in detecting local clusters and has higher power than other available spatial statistical methods [2, 15]. Spatial scan statistical analysis was employed to test for the presence of statistically significant spatial hotspots/clusters [24] of childhood diarrhea using Kuldorff’s SaTScan version 9.4 software [32]. The spatial scan statistic uses a scanning window that moves across study area [26]. Children with diarrhea were taken as cases and without the disease were taken as controls to fit the Bernoulli model. The number of cases in each location had Bernoulli distribution and the model requires data with or without a disease.

The default maximum spatial cluster size of <50% of the population was used, as an upper limit, which allowed both small and large clusters to be detected and ignored clusters that contained more than the maximum limit. For each potential cluster, a likelihood ratio test statistic was used to determine if the number of observed diarrhea cases within the potential cluster was significantly higher than expected or not. The primary and secondary clusters were identified and assigned *p*-values and ranked based on their likelihood ratio test, on the basis of 999 Monte Carlo replications [17, 33].

## Results

### Trends and distribution of childhood diarrhea

**Fig. 2:**
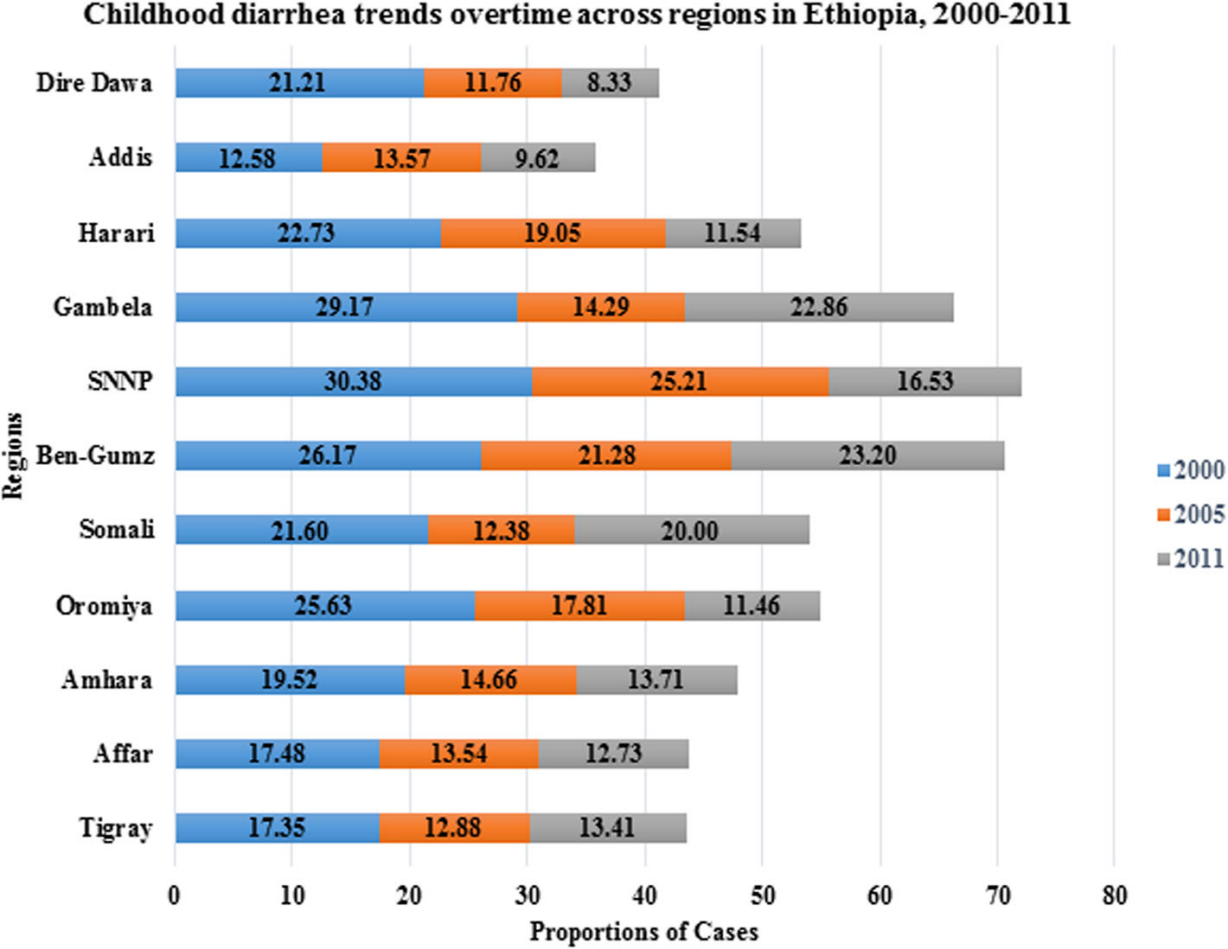
Childhood diarrhea trends overtime across Regions in Ethiopia, 2000, 2005 and 2011. This stacked bar chart shows the magnitude of childhood diarrhea observed among regions with their time trends. The numbers labelled on each bar shows diarrhea prevalence (%). Its magnitude was reduced from 2000 to 2005 in all regions, but increased from 2005 to 2011 in Gambella, Benshangul-gumuz, Somali, and Tigray regions

**Fig. 3:**
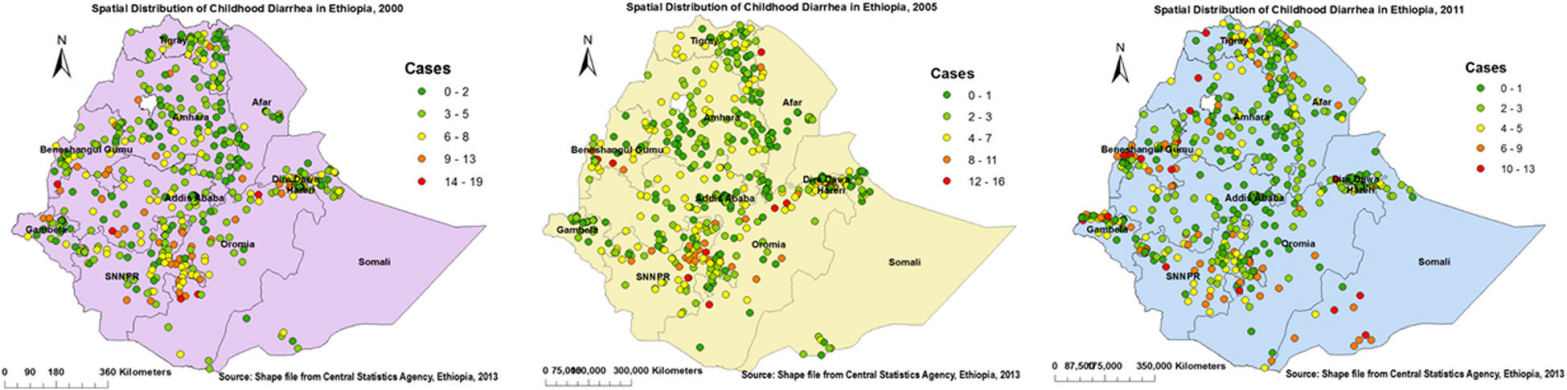
Spatial distribution of Childhood Diarrhea cases across Regions in Ethiopia, 2000, 2005, and 2011. - On this multi-panel figure, each spot (point data) on the map represents one census enumeration area which encompasses a number of diarrhea cases. The more cases it contains show diarrhea hotspot areas. The red color indicates areas with high rates of diarrheal cases whereas the green one indicates low rates of diarrhea. The disease rates were decreased from time to time sequentially

Overall, childhood diarrhea shows the decreasing pattern in the country, however, not consistently decreased across the regions (Fig. 2). Generally, a slight decline trend of childhood diarrheal cases was observed since 2000 (Fig. 3).

### Spatial patterns of childhood diarrhea

**Fig. 4:**
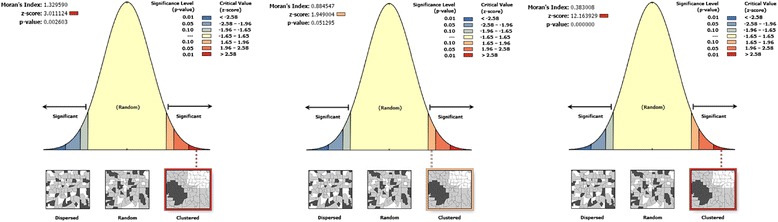
Spatial Patterns of childhood diarrhea in Ethiopia, 2000, 2005, and 2011. The clustered patterns (on the right sides) show high rates of diarrhea occurred over the study area. The outputs have automatically generated keys on right and left sides of each panel. Auto-generated interpretations available underneath each panel show that the likelihood of clustered patterns occurred by random chance are less than 1%. The bright red and blue colors (to the end tails) indicate increased significance level

The spatial patterns of childhood diarrhea were found non-random at all study periods (Fig. 4). The Global Moran’s I values (0.38–1.33) indicated that there was significant clustering of childhood diarrhea in the country. The clustering pattern of 2000 and 2011(>99%) were significantly different as compared to in 2005 (>90%) (Fig. 4-plot 2, Table 2).

**Table 2:** Spatial autocorrelation analysis of childhood diarrhea in Ethiopia, 2000, 2005, and 2011

EDHS Study periods	Observed Moran’s I	Expected Moran’s I	Z-Score	*P*-value
2000	1.3296	−0.0019	3.01	0.001
2005	0.8845	−0.0019	1.95	0.05
2011	0.3830	−0.0018	12.16	0.001

The table shows that when the observed value is greater than the expected value and *P*-value < 0.05, it is statistically significant

### Spatial epidemiology of childhood diarrhea

**Fig. 5:**
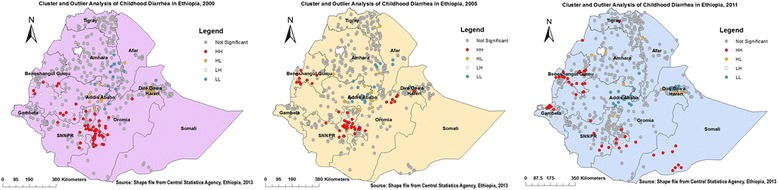
Cluster Outlier identification of childhood diarrhea in Ethiopia, 2000, 2005, and 2011. Each point on the map represents a single enumeration area with a number of diarrhea cases. HH (High-High) means high rates of diarrheal cases surrounded by similar characteristics; HL (High-Low) means high rates of diarrheal cases surrounded by low rates of diarrheal cases; LH (Low-High) means low rates of diarrheal cases surrounded by high rates of diarrheal cases; and LL (Low-Low) means low rates of diarrheal cases surrounded by similar characteristics. The red (HH) color indicates diarrhea hotspot areas, the blue (LL) color indicates diarrhea cold spot areas, and the yellow (HL) and white (LH) colors indicate outliers. The hotspot areas are public health important

**Fig. 6:**
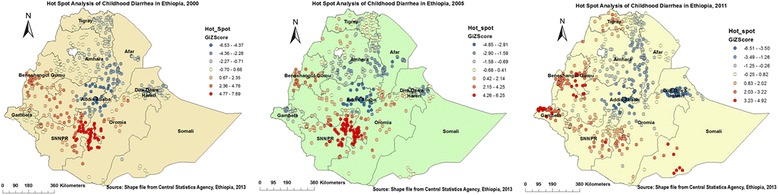
Hot spot identification of childhood diarrhea in Ethiopia, 2000, 2005, and 2011. Dark red colors show significant (*p*-value < 0.001) clusters of diarrheal cases (risk areas), whereas, dark blue colors show significant (*p*-value < 0.001) non-risk areas. The more clustered red and blue colors indicate more diarrhea risk and non-risk areas, respectively. When the Z-score increases (+/−), its significance level increase

Figures 5 and 6 indicate the geographical distribution of childhood diarrhea. The hot spot regions were SNNP and Oromia (all panels); Benshangul-gumuz (middle and right); Harari (middle); Somali, Gambella, north-west Amhara, and Central Tigray (right panel) regions. Whereas, the two town administrations (Addis Ababa, Dire Dawa), central Oromia and eastern Amhara (all panels); and Harari (left and right panels) regions were indicated as cold spot regions. The outliers were found on Addis Ababa and Dire Dawa (left); Oromia, Harari, Amhara, Benshangul-gumuz, and SNNP (all panels); Afar (left and right); Gambella and Somali (left) regions.

### Spatial interpolation

**Fig. 7:**
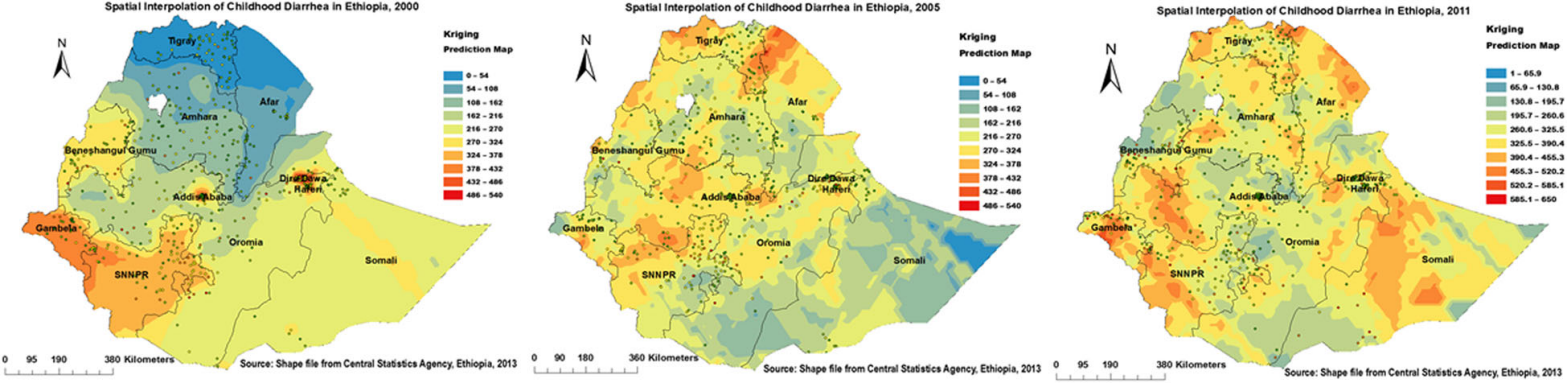
Interpolated spatiotemporal trends of childhood diarrhea in Ethiopia, 2000, 2005, and 2011. Continuous images produced by interpolating (Kriging interpolation method) diarrheal cases among childhoods in 2000, 2005, and 2011. The dark red ramp color indicates the predicted diarrhea high risk areas and yellow ramp color indicates less risk areas of diarrhea

The red prediction areas show predicted risk regions and the children living in those quarters were vulnerable to childhood diarrhea. In the first panel, Gambella and SNNP regions were predicted as more risky areas compared to other regions. In the middle panel, the northern Afar, Central Oromia and SNNP, the northern and eastern border areas of Tigray and western Amhara regions were identified as risk areas. In the right panel, the prediction has gone to the border areas of SNNP, Tigray, and Afar regions; Central Somali and western Oromia, Gambella and Amhara (West Gojam, Awi, Oromia, and Wag Himra zones) regions. These were predicted as risk areas for childhood diarrhea (Fig. 7).

### Spatial scan statistical analysis

**Fig. 8:**
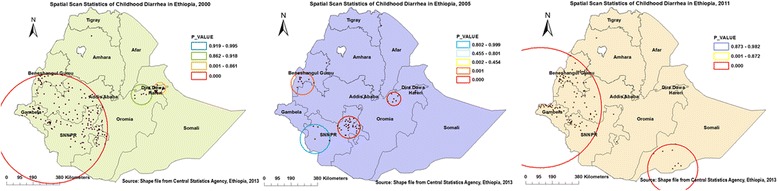
Most likely and secondary clusters of childhood diarrhea in Ethiopia, 2000, 2005, and 2011. The bright red colors (rings) indicate the most statistically significant spatial windows which contain primary clusters of diarrhea. Though the biggest spatial windows (in right and left panels) extended beyond the study area, they do not contain any data outside the boundary. Because by default the spatial window centered enumeration area (cluster number) with the highest number of cases to draw the ring radius. Interpretation: Childhoods within the spatial window (cluster) have higher risk of diarrhea than childhoods outside the spatial window

A total of 337 significant clusters (165 in 2000, 50 in 2005, and 122 in 2011) were identified. Of which, 311 were most likely (primary) clusters and 26 were secondary clusters (18 in 2005 and 8 in 2011). In 2000, the only big spatial window was located in Western and South Western (Gambella, SNNP, Western parts of Oromia and south parts of Benshangul-gumuz) regions of Ethiopia. The clusters spatial window was centered at 6.888619 N, 35.420836 E with 398.0 km radius, with a relative risk (RR) of 1.5 and Log-Likelihood ratio (LLR) of 55.4, at *p* < 0.001. Which means children within the spatial window had 1.5 times higher risk of diarrhea than children outside the window (Table 3, Fig. 8-left panel).

**Table 3:** Significant spatial clusters of childhood diarrhea EDHS 2000, 2005 and 2011, Ethiopia

Years	Clusters	Enumeration areas (clusters) detected	Coordinates/radius	Population	Cases	RR	LLR	*P*-value
2000	1	375, 376, 384, 413, 419, 412, 409, 410, 407, 411, 406, 405, 408, 371, 370, 404, 374, 372, 401, 373, 174, 400, 391, 180, 351, 359, 183, 395, 397, 179, 399, 368, 394, 181, 173, 390, 388, 360, 393, 178, 385, 418, 417, 415, 392, 416, 171, 389, 369, 398, 365, 386, 396, 182, 165, 172, 403, 414, 177, 236, 361, 387, 358, 362, 402, 357, 164, 383, 364, 366, 170, 363, 377, 367, 353, 311, 161, 379, 163, 326, 352, 334, 175, 356, 162, 331, 380, 378, 328, 354, 332, 323, 325, 355, 322, 169, 176, 307, 315, 321, 320, 333, 337, 186, 160, 310, 309, 338, 316, 348, 166, 341, 314, 187, 349, 167, 303, 344, 343, 350, 342, 340, 382, 335, 308, 381, 292, 305, 336, 300, 185, 319, 232, 324, 235, 168, 302, 191, 237, 318, 230, 298, 233, 317, 240, 299, 202, 345, 192, 201, 339, 296, 295, 297, 188, 293, 346, 304, 306, 313, 347, 291, 301, 200, 231	(6.888619 N, 35.420836 E)/398.0 km	3150	930	1.50	55.37	<0.001
2005	1	537, 46, 464, 517, 292, 12, 126, 224, 434, 35, 45, 202, 105, 504, 252, 340, 343, 279, 514, 534, 80, 13, 30, 235, 67, 40, 7, 210, 380, 168, 436, 229	(6.973082 N, 37.814081 E)/80.3 km	616	200	2.01	45.69	<0.001
	2	100, 486, 33, 247, 6, 290, 242	(8.830199 N, 40.729640 E)/49.1 km	171	62	2.15	18.01	<0.001
	3	107, 118, 305, 144, 518, 254, 34, 251, 5, 520, 480	(9.822890 N, 34.781552 E)/82.1 km	223	70	1.86	13.62	<0.01
2011	1	206, 339, 116, 375, 267, 434, 354, 30, 545, 125, 107, 468, 61, 294, 586, 432, 160, 511, 368, 223, 639, 631, 395, 327, 559, 641, 563, 317, 443, 332, 40, 490, 264, 329, 100, 625, 389, 449, 505, 404, 276, 315, 142, 349, 457, 6, 341, 157, 562, 207, 409, 364, 204, 475, 622, 642, 619, 243, 324, 112, 607, 578, 477, 411, 209, 520, 277, 259, 438, 118, 232, 598, 269, 136, 390, 405, 367, 113, 166, 58, 302, 580, 253, 54, 623, 193, 135, 91, 94, 283	(8.219330 N, 33.321854 E)/435.8 km	2170	483	1.69	51.09	<0.001
	2	266, 295, 48, 381, 250, 240, 346, 627	(4.240002 N, 41.906017 E)/179.5 km	216	73	2.30	23.94	<0.001

A cluster is statistically significant when its LLR is greater than the critical value, which is, for significance level: (Standard Monte Carlo Critical Values: 0.001: 18.73; 0.01: 10.89; 0.05: 8.82)^2000^, (0.001:14.17; 0.01: 10.99; 0.05: 8.98)^2005^, (0.001:15.14; 0.01: 10.91; 0.05: 9.32)^2011^)

The small sized spatial window was located in SNNP region, while the secondary clusters spatial windows were located in Benshangul-gumuz and northeast Oromia regions. The primary clusters spatial window was centered at 6.973082 N, 37.814081 E with 80.3 km radius, with a RR of 2.0 and LLR of 45.7, *p* < 0.001. Whereas the secondary clusters spatial window was centered at 8.830199 N, 40.729640 E with 49.1 km radius, with a RR of 2.2 and LLR of 18.0, at *p* < 0.001 (Table 3, Fig. 8-middle panel).

Only two spatial windows were plotted. The biggest primary clusters spatial window was typically located in the Western edge of the country, which encompasses Gambella, Western parts of Oromia, SNNP and Benshangul-gumuz regions. Whereas, the secondary clusters spatial window was located in southwest part of Somali region. The primary clusters spatial window was centered at 8.219330 N, 33.321854 E with 435.8 km radius, with a RR of 1.7 and LLR of 51.1, at *p* < 0.001. While the secondary clusters spatial window was centered at 4.240002 N, 41.906017 E with 179.5 km radius, with a RR of 2.3, and LLR of 23.9, at *p* < 0.001 (Table 3, Fig. 8-right panel).

## Discussion

Our study indicates that childhood diarrhea at national and regional levels are non-random. Significant clusters were detected in SNNP, Oromia, Benshangul-Gumuz, Harari, Somali, and Gambella in 2000 and 2005, and Benshangul-Gumuz, Gambella, and Somali regions in 2011. Diarrhea risk map of 2011 revealed that the border areas of SNNP, Tigray, and Afar, Central Somali and West Oromia regions were risk areas of diarrhea transmission. A total of 165 significant clusters found in Western parts of the country (2000); fifty significant clusters found in SNNP, Oromia, and Benshangul-Gumuz in 2005; and 122 clusters found in Gambella, Benshangul-Gumuz, West Oromia and SNNP, and Somali regions in 2011.

The trend of childhood diarrhea (Fig. 2) showed a declined pattern consistent with a study conducted in northwest Ethiopia [2]. This could be the fact that a lot of works were done on the improved Water, Sanitation, and Hygiene (WASH)-related interventions carried out by the government and non-governmental organizations [34]. The contribution of health extension workers on the improvement of child health was paramount [35]. However, because of environmental and hygiene related packages are sustainable, the incidence of childhood diarrhea observed low in model graduated households [10, 36–38]. This study exhibited the increasing trends of diarrhea in Gambella, Benshangul-gumuz, and Somali regions (Fig. 2) which are located in geographically remote areas. This is because of the fact that adequacy, availability and accessibility of safe water is not satisfactory in all regions of the country especially, in the remote and rural areas.

The 2000 and 2011 datasets of spatial autocorrelation analysis revealed that diarrhea had high spatial dependency (respectively, Moran’s I: 0.38 and 1.32, .01) in agreement with studies done in Thailand and Malawi [5, 39]. Heterogeneity of disease clustering found across Oromia, SNNP, Benshangul-gumuz, Harari, Somali, Gambella, northwest Amhara, and Central Tigray regions. The high rates of diarrhea morbidity showed in 2000 and 2005 was shifted from SNNP region to Gambella, Benshangul-gumuz, and Somali regions. This might be due to WASH-related interventions carried out by government and non-governmental organizations in SNNP region [36]. Both in hotspot and cluster/outlier analyses, the high risk regions were identified consistently and they may be vulnerable to diarrhea outbreaks.

In smoothing diarrhea risk map, the border areas of SNNP, Tigray, and Afar, Central Somali and Western Oromia, and Amhara (West Gojam, Awi, Oromia, and Wag Himra zones) were estimated as childhood diarrhea risk areas. These areas might be more vulnerable to diarrhea morbidity due to inaccessibility of health services, shortage of safe and adequate drinking water supply, low altitude (lowland) areas where malaria endemic which hasten the infection [40].

In 2000 survey, the spatial scan statistics detected 165 significant clusters which could help policy-makers to make decisions at regional levels. However, this large spatial clusters window covered a large area with a larger and more heterogeneous population (Fig. 8-left panel). In 2005, Moran’s I analysis revealed marginally significant clusters and statistically significant clusters detected in SaTScan evaluation. This might be due to the power evaluation differences in between the two tests [15]. A spatial window with 32 primary clusters at SNNP region was identified as hotspots, which was typically alike to the results revealed by both Anselin Local Moran’s I and Gettis-Ord G* statistical analysis. This might show that the findings are valid and consistent.

In 2011, the purely spatial analysis detected 114 most likely and 8 secondary significant clusters which were found in Gambella, Benshangul-gumuz, Western Oromia and SNNP, and Somali regions. High rates of childhood diarrhea identified almost in neighboring regions which share similar geographical parameters and culture. This might be due to that those regions are located geographically in lowland areas where there is high rates of co-wives which were determinant factors for childhood diarrhea in the regions (Gebre G, Alemu K, et al. 2016, Determinants of childhood diarrhea in Ethiopia: data from Ethiopia Demographic and Health Surveys (2000–2011), unpublished).

In this study data were representative at the national, regional, and rural-urban levels and can be generalized to all childhoods in Ethiopia. The Geographic information system (GIS) and SaTScan statistical tests detected similar and statistically significant high-risk clusters/hotspots of diarrhea. The visualization and cluster analysis can provide valuable information about the spatial disparity of childhood diarrhea which may warrant for further research. However, the location data values were shifted 1-2kms for urban and 5kms for rural areas for data confidentiality issues, consequently, this was the challenge to know the exact cases’ location. The data used for this study were not taking into account seasonal variations of childhood diarrhea. The DHS surveys did not base on clinically confirmed data, rather rely on the mothers’/ caregivers’ report. Mothers from different backgrounds are likely to have different perception of childhood illness. Accordingly, under or over reporting of the illness may be occurred.

The findings of this study has valuable policy implications for health programs design and interventions. The diarrhea hotspot areas can be easily identified even at district/kebele level to take local interventions. It may also be important to prevent and control suspected diarrhea outbreaks. In general, these findings supreme important for Ministries of Health and Water, Health Bureaus, and partners to develop interventions programs against childhood diarrhea.

## Conclusion

In this study, childhood diarrhea remains public health problem and had a spatial variation across the regions. Though a declining pattern of diarrhea was shown at the national level, the morbidity remained high in regions, such as Gambella, Benshangul-gumuz, and Somali. Findings suggest that priority attentions would be important on water, sanitation, and hygiene-related interventions on the identified hotspot areas to prevent and control diarrhea. Child’s postnatal check with 2 months after birth, effective educational program against polygamy and women education would be important.

## Abbreviations

DHS: demographic and health survey
EA: enumeration area
EDHS: Ethiopia demographic and health survey
GIS: geographic information system
LLR: Log likelihood ratio
SNNP: Southern Nations Nationalities and Peoples Region
WASH: water, sanitation, and hygiene
